# Sex-Specific Effects of Early-Life Stress Exposure on Memory Performance and the Medial Prefrontal Cortex Transcriptomic Pattern in Adolescent Mice

**DOI:** 10.1007/s12035-025-04803-x

**Published:** 2025-03-04

**Authors:** Rodrigo Orso, Thiago Wendt Viola, Bernardo Aguzzoli Heberle, Kerstin Camile Creutzberg, Francisco Sindermann Lumertz, Rodrigo Grassi-Oliveira

**Affiliations:** 1 Department of Psychiatry, University of Colorado Anschutz Medical Campus, Aurora, USA; 2 School of Medicine, Pontifical Catholic University of Rio Grande Do Sul, Porto Alegre, Brazil; 3 Sanders-Brown Center on Aging, University of Kentucky, Lexington, USA; 4 Translational Neuropsychiatry Unit, Department of Clinical Medicine, Aarhus University, Palle Juul-Jensens Boulevard 11, A701-129, 8200 Aarhus, Denmark

**Keywords:** Maternal Separation, Limited Bedding, RNA-seq, Working Memory, Short-Term Memory

## Abstract

Early life stress (ELS) is considered a risk factor for the development of cognitive and executive dysfunctions throughout development. The medial portion of the prefrontal cortex (mPFC) is directly implicated in short-term working memory. Furthermore, due to its late development compared to other brain regions, the mPFC is considered a vulnerable brain region to ELS exposure. Here, we investigated the effects of the ELS on PFC-dependent memory and mPFC transcriptomic profiles. From postnatal day (PND) 2 to PND 15, BALB/cJ mice were exposed to maternal separation (MS) for 3 h per day combined with limited bedding (ELS group) or left undisturbed (CT group). During the period of stress, maternal behavior was recorded pre-MS and post-MS. From PND 45 to PND 47, males and females were tested for working memory performance in the Y-maze and short-term recognition memory in the object in place task (OIP). Later, we assessed mRNA level alterations in the mPFC by RNA-seq. Here, we showed that ELS increases maternal care post-MS and the number of nest exits pre-MS and post-MS. Furthermore, males and females exposed to ELS exhibited impairments in the OIP, while only females performed worse in the Y-maze. With respect to the mPFC transcriptome, we identified 13 DEGs in the females, which were significantly influenced by chaperone-mediated protein folding processes, while 4 genes were altered in males. In conclusion, we showed that, compared with male sex, ELS alters maternal behavior and leads to more extensive impairments in memory function and transcriptomic alterations in females.

**Supplementary Information:**

The online version contains supplementary material available at 10.1007/s12035-025-04803-x.

## Introduction

Early life stress (ELS) is considered a risk factor for the development of several psychiatric conditions [1], as is increased vulnerability to cognitive and executive dysfunctions later in life [2]. Moreover, long-term alterations in the hypothalamic‒pituitary‒adrenal (HPA) axis response and impairments in the corticolimbic system circuitry have been strongly associated with adverse experiences during early development [3, 4]. The prefrontal cortex (PFC) is one of the brain regions included in the corticolimbic system, and mainly, its medial portion (mPFC) is directly implicated in short-term and working memory, decision-making, and emotion regulation [5]. Due to its late development compared to that of other brain regions and high density of glucocorticoid receptors, the mPFC is considered a vulnerable brain region to the effects of ELS [6]. Previous studies have reported that exposure to preclinical models of ELS can impair memory performance on PFC-dependent tasks [7–9]. Nevertheless, there are still gaps in the literature to fully unravel the molecular underpinnings associated with those long-term cognitive deficits. In addition, considering the polygenic characteristics of these impairments, a genome-wide approach is increasingly necessary to comprehend such conditions.

Recent unbiased transcriptome analyses of the mPFC in rodents exposed to ELS have yielded diverse findings. For instance, Shi et al. [10] reported a heightened vulnerability of the oxytocinergic gene pathway to postnatal maternal separation (MS) in rats, whereas Yang et al. [11] reported that MS adversely affects dopamine signaling and neuronal development pathways in the mPFC of C57BL/6 J mice. Other studies combined the ELS paradigm with stress exposure during adulthood and revealed significant alterations in gene pathways such as extracellular matrix structure, collagen trimers, and ribosomal pathways in the mPFC [12, 13]. Furthermore, Peña et al. [14] demonstrated sex-specific effects within an ELS model that combined limited bedding (LB) and MS, occurring from postnatal day 10 to 17 in females and from postnatal day 10 to 20 in male C57BL/6 J mice. Their findings indicated that in males, ELS predominantly impacted the myelination pathway in the mPFC, while in females, it primarily affected immune system processes. These findings underscore the influence of rodent strain, sex, and stress model on transcriptome experiments investigating the enduring consequences of ELS. As a result, differentially expressed genes and pathways affected in the mPFC can vary substantially across studies. All the aforementioned studies analyzed transcriptome changes induced by ELS in adult animals. However, it is noteworthy that adolescent animals may exhibit specific alterations, both in molecular and behavioral phenotypes, in response to ELS compared to adult animals [15]. This distinct age factor introduces another layer of complexity to the understanding of the long-term effects of ELS in rodent models. In adolescent animals, males may exhibit greater vulnerability to PFC dysfunction following ELS than females [16]. However, this conclusion was drawn without the use of a genome-wide investigation, highlighting the necessity of an unbiased transcriptomic approach.

In this study, we aimed to explore the effects of the ELS on working and short-term recognition memory and on the mPFC transcriptomic profiles of male and female adolescent mice. Our research addresses a significant gap in animal studies, where the majority of ELS studies investigate adult animals. Furthermore, adolescence is a critical period of heightened vulnerability during which the effects of ELS are likely more pronounced, particularly in brain regions that undergo later development. Unlike previous studies in which transcriptomic analyses were conducted in this region, we concentrated our investigation on BALB/cJ mice, a strain known for its heightened sensitivity to stress [17].

## Methods

### Animals

This study was performed with male and female BALB/cJ mice obtained from the Center of Experimental Biological Models (CeMBE) at Pontifical Catholic University of Rio Grande do Sul (PUCRS), Porto Alegre, Brazil. All animals were housed in mouse Plexiglas cages (22 cm × 16 cm × 14 cm) with controlled ventilation, temperature (21 °C ± 1 °C) and humidity (55% ± 5%). A 12/12 h light–dark cycle was implemented (lights on at 7 a.m. to 7 p.m.), with mouse chow and water available ad libitum. All experiments were approved by the Ethics Committee on Animal Use (CEUA) of PUCRS under #8546 and conducted in accordance with the Guide for the Care and Use of Laboratory Animals (NIH).

**Fig. 1:**
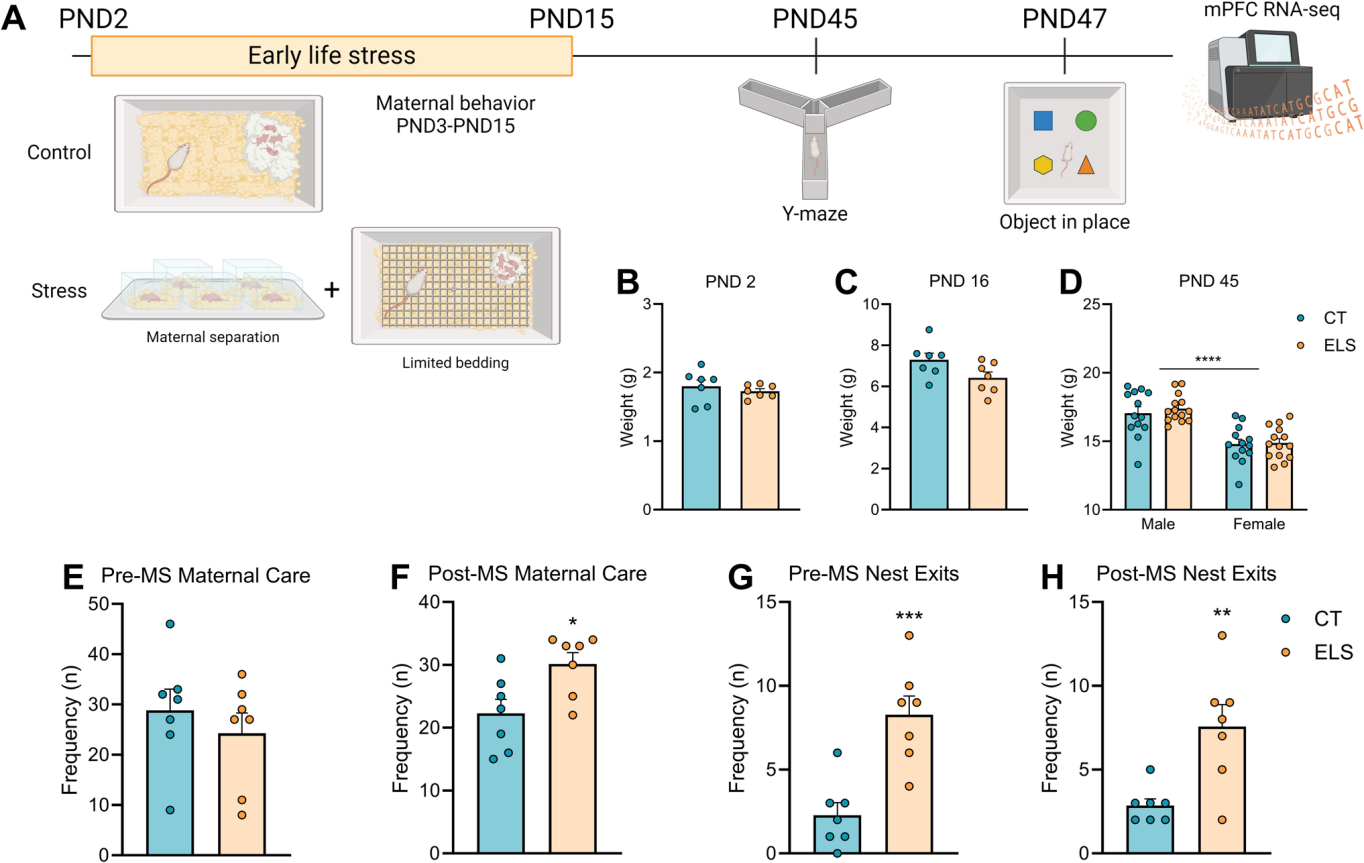
Experimental timeline, body weight, and maternal behavior analysis (**A**) Experimental design; (**B-C**) PND 2 and PND 16 represent the whole litter weight divided by the number of pups per litter. PND 45 represents the body weight of individual offspring; (**E**) frequency of maternal behaviors before MS; (**F**) frequency of maternal behaviors after MS; (**G**) frequency of nest exits before MS; (**H**) frequency of nest exits after MS. The results are expressed as the mean ± SEM; *n* = 7 litters per group (PND 2, PND 16, and maternal behavior); and *n* = 13–14 animals per group on PND 45. * *p* < 0.05; ** *p* < 0.01; *** *p* < 0.001; **** *p* < 0.0001. (Student’s t test for PND 2, PND16, and maternal behavior; two-way ANOVA for PND 45)

To avoid stress associated with animal transportation, the animals were bred in house. The breeding procedure consisted of housing two females and one male in the same cage for three days. Two weeks later, the females were individually housed and inspected daily until the presence of pups was confirmed. The day of birth was designated postnatal day 0 (PND 0). After birth, cross-fostering was performed, and the litters were culled to 6–8 pups per litter. The dam and pups were then randomly assigned to control (CT) or ELS conditions. For this experiment, we used 7 litters from the CT group and 7 litters from the ELS group. At PND 21, two to three animals per cage were group-housed with same-sex littermates. A maximum of two animals were included per sex for each experimental condition to avoid potential litter effects. A total of 13 male CT, 13 female CT, 14 male ELS and 14 female ELS animals were utilized in this study. The animals were briefly weighed at PND 2, PND 16 and PND 45. At PND 2 and PND 16, the entire litter was weighed, and the total weight was divided by the number of pups in each litter. At PND 45, the animals were weighed individually. Working and short-term recognition memory performance was assessed from PND 45 to PND 47. The experimental timeline can be found in Fig. 1A.

### Early-Life Stress Protocol

The ELS protocol consisted of simultaneously exposing the animals to MS and LB. Our combined ELS protocol was adapted from the model previously proposed by Peña et al. [18], and it is intended to impose multiple adverse experiences during early development. From PND 2 to PND 15, animals from the ELS group were reared in LB cages with a reduced amount of nesting material (1 g of cotton). In addition, the home cage floor was covered using an aluminum mesh platform with wood shavings underneath. The dams could not reach the wood shavings for use as nesting material, but it was necessary to permit the mouse droppings to fall below the platform without trapping the pups. Dams and litters from the CT group were placed in cages with standard amounts of wood shavings and 4 g of cotton to be used as nesting material. After PND 15, animals from the ELS group were returned to regular rearing conditions. Cage cleaning was performed once a week for both groups.

Simultaneously, the MS protocol was performed by separating the pups 3 h per day (9 a.m. to 12 p.m.) between PND 2 and PND 15. To start the protocol, the dams were removed from the home cage and kept in a different colony room to avoid any dam-pup ultrasonic vocalizations. The pups were then individually separated into small plastic containers. A digital-regulated heating pad (32 °C ± 3 °C) was placed underneath the containers to control the temperature and prevent possible hypothermia. After the MS period, the pups were returned to their home cages, followed by delivery via the dam. The CT group was left undisturbed during the protocol.

### Maternal Behavior Analysis

Maternal behavior during the first weeks of development has a direct impact on pup neurodevelopment [19]. For this reason, we conducted intercalated observations of maternal behavior (*n* = 7 dams per group) from PND 3 to PND 15 (7 days total). The observations were performed every 3 min over a period of 18 min (7 observations) before (8:30 a.m.) and after the MS period (2 p.m.). The maternal behaviors evaluated were nursing (N), licking (L), nest building (B) and contact with pups (C). Moreover, during the 18 min of observation, the frequency of exits from the nest performed by the dam was determined. The sum of all maternal behaviors (MB = N + L + B + C) evaluated throughout the days was used for pre-MS and post-MS maternal behavior analysis.

### Y-maze

Working memory performance was evaluated at PND 45 in the Y-maze test. The apparatus consisted of three identical arms (30 cm × 5 cm × 10 cm). This working memory test is performed in two phases (sample and test), and it is based on the premise that rodents are more prone to exploring novel areas than previously explored environments are. In the sample phase, the animals were allowed to explore two arms (the start arm and familiar arm) for 5 min, while the third arm (the novel arm) was blocked with a removable wall. After the sample phase, the animals were kept in a holding cage for 1 min, after which the wall that blocked the novel arm was removed. In the test phase, the animals were allowed to acclimate to the start arm and could freely explore all three arms of the apparatus for 2 min. The apparatus was cleaned with 70% ethanol between animals to avoid potential odor cues. The working memory index was calculated using the following formula: [time spent in the novel arm/(time spent in the familiar arm + time spent in the novel arm)] × 100. The analysis of the time spent in each arm was performed using Any-Maze software version 4.9 (ANY-Maze, Inc., Greensburg, PA).

### Place Object

The object in place (OIP) task was performed at PND 47 in an open field arena (33 cm × 33 cm) to investigate short-term recognition memory. The OIP is performed in two phases (acquisition and test) and requires the animal to associate the characteristics of an object and the spatial location where this object was encountered. One day before the task, the animals were habituated to the apparatus for 10 min. The total distance traveled was evaluated as a measure of locomotor activity during habituation. For the acquisition phase, the animals were introduced to the center of the apparatus, which had 4 different objects in the corners, and were allowed to explore all the objects for 5 min. After the acquisition phase, the animals were placed in a holding cage for 5 min, after which the position of two of the four objects was inverted. In the test phase, the animals were allowed to explore all the objects for 3 min. A recognition index of the time spent exploring the objects that changed position in relation to the objects that were not changed was calculated with the following formula: [time exploring inverted objects/(time exploring intact objects + time exploring inverted objects)] × 100. Object exploration was defined as actively touching the head, sniffing, or pawing the objects. If an animal climbed on top of the objects, it was not considered exploratory behavior [20]. The apparatus and the objects were cleaned with 70% ethanol between each phase and the animals to avoid any olfactory cues. Analysis of exploration time was performed using Any-Maze software version 4.9 (ANY-Maze, Inc., Greensburg, PA).

### Total RNA Extraction and RNA-seq Library Preparation

Two hours after behavioral testing, the animals were euthanized by decapitation without anesthesia, and the entire brain was removed. The mPFC was immediately hand dissected and snap frozen with isopentane on dry ice. Total RNA (*n* = 6/group) was extracted from mPFC tissue homogenates using QIAzol (Qiagen) according to the manufacturer's protocol and reconstituted in 15 µL of RNAse-free water. All RNA samples were quantified via Qubit HS RNA assays (Thermo Fisher Scientific) and had an RNA integrity number (RIN) > 6 as assessed with an Agilent 2100 Bioanalyzer total RNA assay (Agilent Technologies). Total RNA (1 µg) was used to construct 150 bp paired-end (PE) RNA-seq libraries with an Illumina TruSeq™ RNA sample prep kit (Illumina, USA) according to the manufacturer's instructions. The size and concentration of the PE RNA-seq libraries were verified on an Agilent 2100 Bioanalyzer and sequenced on an Illumina NovaSeq 6000.

### Transcriptomic Analysis

Quality control and adapter trimming of the raw data were performed using the wrapper Trim Galore version 0.6.6 [21], FastQC version 0.11.5 [22], and Cutadapt version 3.4 [23]. Quality control statistics were summarized using MultiQC version 1.11 [24]. The *Mus musculus* reference genome and annotations were obtained from the Ensembl Genome Browser (Mus_musculus—Ensembl Genome Browser 104, n.d.). The “fasta” file for the primary assembly of GRCm39 release 104 was used as the reference genome, and the corresponding “gtf” file was used for annotation. Trimmed paired-end Illumina reads were aligned utilizing the STAR aligner version 2.7.9a [25]. Aligned reads were quantified using FeatureCounts version 2.0.1 [26]. Quality control, adapter trimming, alignment, and quantification steps were implemented as part of a pipeline using the Nextflow programming language version 21.04.3 [27]. The Nextflow pipeline was implemented using a Singularity container [28]. Nextflow in combination with singularity containers enables reproducible computational workflows across platforms. RNA-seq outlier samples were detected utilizing the PcaGrid function [29].

On average, 87% of the reads were successfully aligned, resulting in an average of 19.7 million uniquely aligned reads per sample. The removal of 1 outlier per group from downstream analysis resulted in 5 biological replicates per condition. Differential gene expression (DGE) analysis was performed utilizing DeSeq2 version 1.34.0 [30]. Using Benjamini–Hochberg false discovery rate correction, the thresholds for significance in the DGE analysis were an adjusted p value < 0.1 and a 2logFC ± 0.5. A Gene Ontology (GO) analysis was performed for the genes with an unadjusted p value < 0.01 using the gProfiler2 R package. Enriched pathways (adjusted p value < 0.05) were selected for biological or molecular process enrichment. The graphical representation of the transcriptomic findings was generated using the ggplot2, enhancedvolcano, and enrichplot R packages.

The deconvolution of RNA-seq data to estimate cell-type proportions in each sample was conducted using the CIBERSORT R package. The reference dataset for this analysis was the Allen Brain Map Mouse Cortex and Hippocampus SMART-seq single-cell RNA sequencing data. This reference included profiles of astrocytes, microglia, oligodendrocytes, pyramidal neurons, and interneurons.

### Validation of Transcriptomic Data

Real-time quantitative polymerase chain reaction (qPCR) was performed in a distinct cohort of animals exposed to the same ELS model to validate the RNA-seq results. Total RNA (500 ng) from the mPFC was utilized for complementary DNA (cDNA) synthesis using the QuantiTect Reverse Transcription Kit (Qiagen) according to the manufacturer's instructions. Real-time qPCR was performed using a Rotor Gene (Qiagen), and each SYBR Green PCR was run in duplicate. The relative fold change in expression was calculated using the ΔΔ Ct method, with the control group of the same sex serving as a reference and the Pgk ct values serving as endogenous controls. The sequences of primers used are listed in Table S1. To verify primer specificities, melting curve analyses and agarose gel electrophoresis were performed.

### Statistical Analysis

Two-way ANOVAs were used to analyze group differences according to rearing condition (ELS and CT) and sex (male and female). When necessary, ANOVAs were followed by Tukey’s post hoc tests to investigate specific effects in pairwise comparisons. The normality of the data distribution was analyzed for all variables using the Shapiro‒Wilk test of normality, and the homogeneity of variance was tested via Levene’s test of equality of error variances. The results are expressed as the group mean ± standard error of the mean (SEM), with individual subjects represented with dots, and a p value < 0.05 was considered to indicate statistical significance. Statistical analyses were performed with SPSS software v.25.0 (SPSS, Chicago, IL, USA) and GraphPad Prism 9 (GraphPad Software, Inc., La Jolla, CA, USA).

## Results

### Body Weight Control

At PND 2, no significant difference in weight was observed between the groups before the start of the ELS protocol (Fig. 1B). One day after the end of the ELS (PND 16), we observed a trend in which animals exposed to the ELS had a reduction in weight [t (12) = 2.051, *p* = 0.062; Fig. 1C]. At PND 45, females showed an expected reduction in body weight compared to males, independent of rearing condition [sex effect: F (1,50) = 44.61, *p* < 0.001; Fig. 1D].

### Early-Life Stress Exposure Disrupts the Quality and Frequency of Maternal Care

Considering the importance of adequate maternal behavior in offspring neurodevelopment, we performed a pre-MS and post-MS maternal care analysis. In relation to the frequency of maternal care, no significant differences between groups were observed pre-MS (Fig. 1E), but post-MS maternal care was significantly increased in ELS dams [t (12) = 2.728, *p* = 0.018; Fig. 1F]. Moreover, ELS dams presented a fragmented pattern of maternal care, which was manifested by an increased frequency of nest exit pre-MS [t (12) = 4.494, *p* < 0.001; Fig. 1G] and post-MS [t (12) = 3.447, *p* = 0.004; Fig. 1H].

### Early-Life Stress Impairs the Working and Recognition Memory of Adolescent Mice

**Fig. 2:**
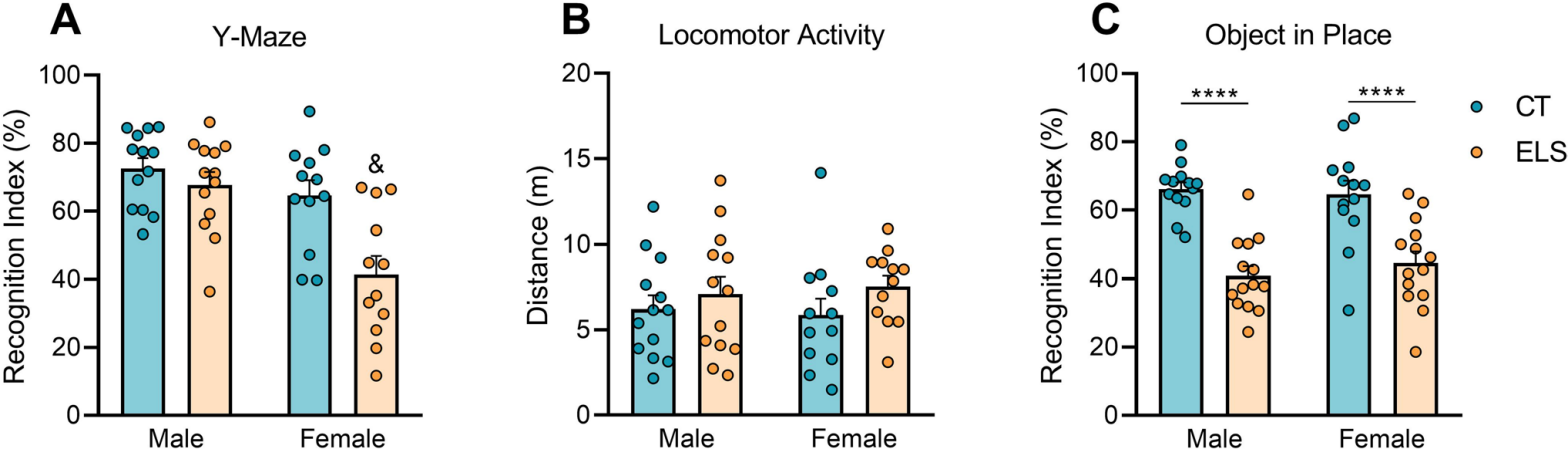
Analysis of working memory, short-term recognition memory, and locomotor activity in male and female adolescent mice. (**A**) Index of working memory in the Y-maze, which takes into account the time spent exploring the novel arm; (**B**) index of recognition in the OIP task, which takes into account the time spent exploring the inverted objects; (**C**) distance explored the apparatus during the habituation phase of the OIP task. The results are expressed as the mean ± SEM; *n* = 13–14 animals per group. **** *p* < 0.0001; & Interaction effect (*p* < 0.05). (two-way ANOVA)

Considering the effects of ELS exposure on PFC-dependent memory function, we investigated the performance of males and females using the Y-maze for working memory and the OIP for short-term recognition memory. Regarding the Y-maze test, we identified a significant interaction effect [F (1, 46) = 4.732, *p* = 0.034; Fig. 2A]. Post hoc analysis revealed that, compared with CT animals, females exposed to ELS presented a reduction in the working memory index (*p* = 0.002), while this effect was not observed in male animals. In relation to the OIP task, we observed that animals from the ELS group interacted significantly less with the inverted set of objects than did the CT animals, independent of sex [F (1,50) = 51.23, *p* < 0.001; Fig. 2C]. No difference in locomotor activity in the habituation phase of the OIP task was observed between the groups (Fig. 2B).

### Early-Life Stress Exposure Leads to Sex-Specific Alterations in the Cortical Transcriptomic Pattern

To investigate the effects of early-life stress (ELS) exposure on the mPFC transcriptome, we conducted RNA-seq analysis on adolescent animals, analyzing males and females separately and postnatal treatment (control or ELS) as a between-subjects factor. Before performing differential gene expression analysis, we deconvoluted the RNA-seq data using publicly available single-cell RNA sequencing data from mouse brains as references. The proportions of astrocytes, pyramidal neurons, microglia, and interneurons were analyzed, and the proportion of oligodendrocytes was found to be negligible. No significant group effects or group-by-sex interaction effects were observed for the cell-type proportions (Figure S1). However, compared with those of male patients, the proportions of astrocyte [F (1, 24) = 5.134, *p* = 0.035] cell types were significantly lower in females, while the proportions of interneuron [F (1, 24) = 6.526, *p* = 0.019] cell types were significantly greater in females than in males, which highlights the importance of performing DGE analysis stratified by sex.

**Fig. 3:**
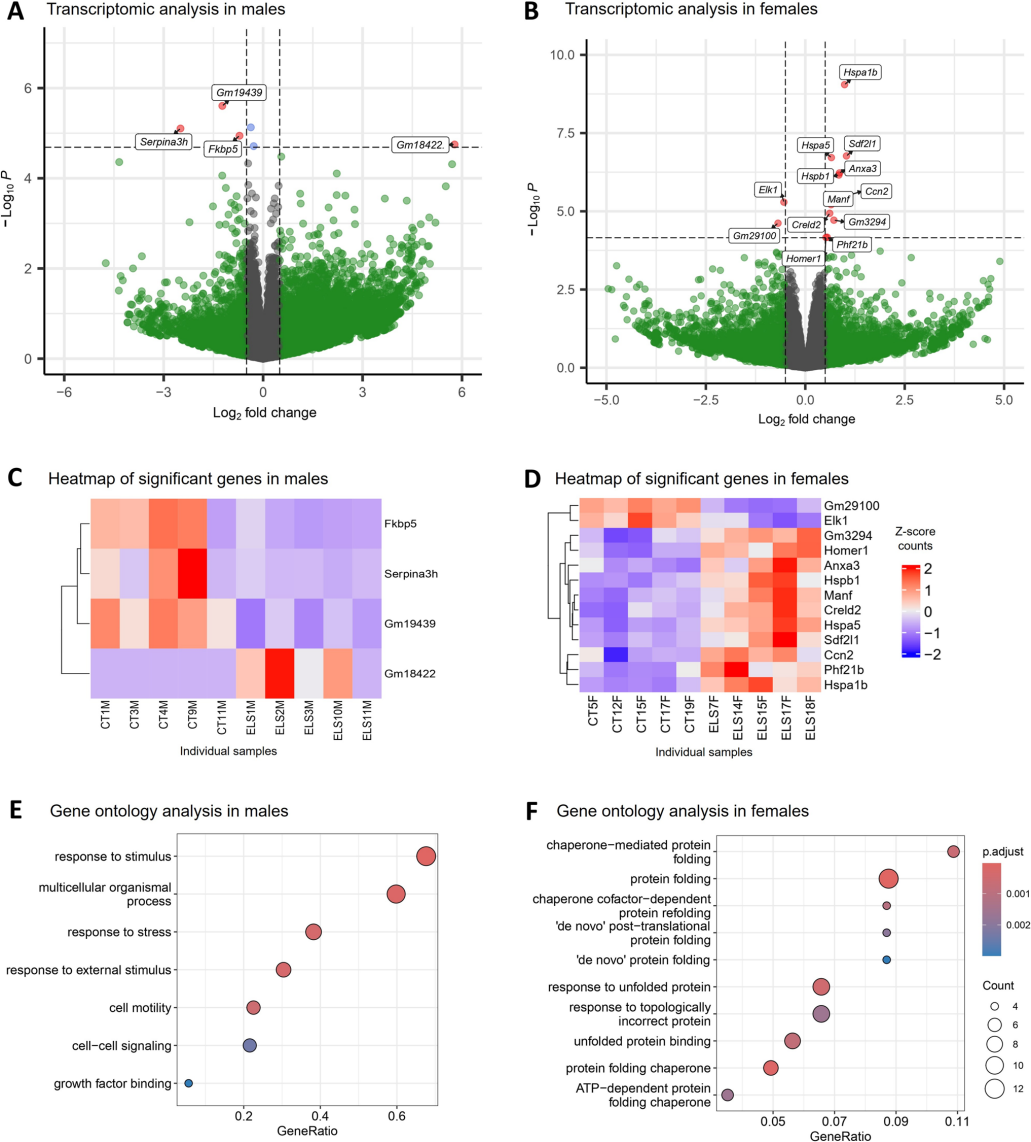
Medial prefrontal cortex transcriptomic analysis of male and female adolescent mice. (**A**) Volcano plot showing DEGs in males; (**B**) Volcano plot showing DEGs in females; (**C**) Heatmap of DEGs in males; (**D**) Heatmap of DEGs in females; (**E**) Top enriched Gene Ontology terms in males; (**F**) Top enriched Gene Ontology terms in females. Differential gene expression: adjusted *p* value < 0.1 and 2logFC ± 0.5. Gene Ontology: unadjusted p value < 0.01. *n* = 5 per group

For males, the analysis revealed 4 DEGs between CT and ELS, three of which were downregulated (*Serpina3h, Fkbp5,* and *Gm19439)* and one (*Gm18422*) of which was upregulated (Fig. 3A). For females, the analysis revealed 13 DEGs, two of which were downregulated (*Elk1* and *Gm29100*), and eleven (*Hspa1b, Hspa5, Sdf2l1, Hspb1, Anxa3, Manf, Ccn2, Creld2, Gm3294, Phf21b* and *Homer1*), which were upregulated (Fig. 3B). The heatmaps show the expression data of the DEGs in the individual samples (Fig. 3C and D). Additionally, a third analysis was conducted to test for potential interactions between group and sex rather than analyzing group effects for each sex independently. However, no significant DEGs were identified in this analysis.

**Fig. 4:**
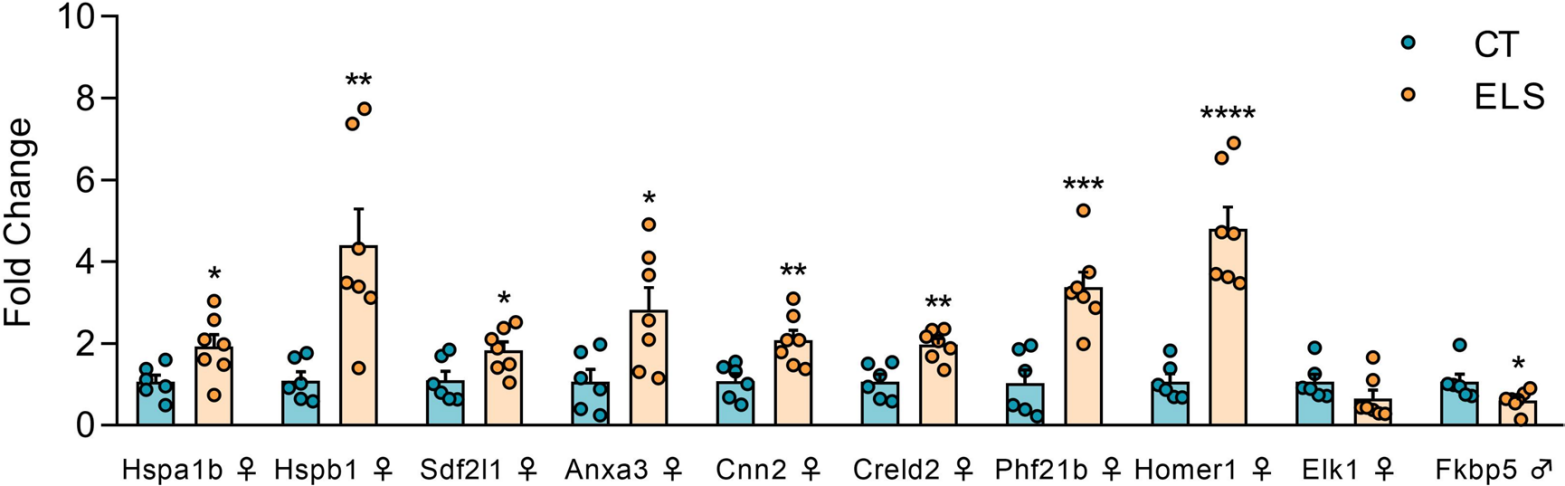
Validation of DEGs following ELS exposure in the mPFC Ten genes identified in the transcriptomic analysis were validated in a separate cohort of males and females using real-time PCR. The results are expressed as the mean ± SEM; *n* = 6–7 animals per group. * *p* < 0.05; ** *p* < 0.01; *** *p* < 0.001; **** *p* < 0.0001. (Student’s t test)

To evaluate the comprehensive impact of ELS exposure on the regulation of the mPFC transcriptome in each sex, we conducted a Gene Ontology (GO) analysis of genes exhibiting differential expression using a less stringent significance threshold (unadjusted p value < 0.01). This analysis revealed enriched pathways associated with chaperone-mediated protein folding in females (Fig. 3F), whereas in males, the enriched pathways were linked to responses to stress and external stimuli (Fig. 3E). To validate the RNA-seq findings, qPCR analyses were performed in a separate cohort of animals exposed to ELS or CT on nine selected DEGs identified in females and one in males. When analyzing the females, we found that ELS exposure induced upregulation of *Hspa1b* [t (11) = 2.520, *p* = 0.028; Fig. 4]; *Hspb1* [t (11) = 3.399, *p* = 0.005; Fig. 4]; *Sdf2l1* [t (11) = 2.435, *p* = 0.033; Fig. 4]; *Anxa3* [t (11) = 2.704, *p* = 0.020; Fig. 4]; *Cnn2* [t (11) = 3.323, *p* = 0.006; Fig. 4]; *Creld2* [t (11) = 4.181, *p* = 0.001; Fig. 4]; *Phf21b* [t (11) = 4.671, *p* < 0.001; Fig. 4]; and *Homer1* [t (11) = 6.221, *p* < 0.001; Fig. 4]. On the other hand, no alteration in *Elk1* gene expression was detected [t (11) = 1.507, *p* = 0.160; Fig. 4]. Among the males, *Fkbp5 was downregulated in the ELS animals* [t (11) = 2.321, *p* = 0.040; Fig. 4].

## Discussion

This study aimed to investigate the effects of a combined model of ELS involving MS and LB on the working and short-term recognition memory performance of adolescent male and female mice, as well as on the mPFC transcriptomic pattern. The main findings of the present study are as follows: (1) Dams exposed to ELSs exhibit an increased frequency of nest exit according to pre-MS and post-MS data and overall maternal care behavior post-MS. (2) Exposure to ELS impaired working memory in females and short-term recognition memory in both male and female adolescent mice. (3) Sex-specific transcriptomic alterations were identified in animals exposed to ELS, where the majority of DEGs were observed in females. (4) Chronomic-mediated protein folding was the top GO term for females, while response to stimulus was the top term observed for males. These findings indicate that females may experience more pronounced memory impairments following ELS exposure and that alterations in chaperone-mediated protein folding processes might be involved in such memory deficits.

Maternal care during the first weeks of development plays a key role in pup neurodevelopment [31, 32]. However, exposure to ELS may disrupt this relationship, which can have a long-term impact on the development of offspring [19]. Here, we showed that ELS increased the frequency of maternal care post-MS, suggesting a potential adaptative response as an attempt to mitigate the effects of MS. Indeed, previous studies have shown that this increase in maternal care may attenuate the long-term impairments provoked by MS [33–35]. Furthermore, previous studies have demonstrated that LB exposure disrupts the quality of maternal care, which is mainly due to an increase in the number of nest exits [8, 36]. This fragmentation was observed in our study via both pre-MS and post-MS analysis. This increase in nest exits may not only prolong the time that dams are away from pups but also interrupt important behaviors, such as nursing and licking [37].

Regarding the performance in the memory tasks evaluated, we identified sex-specific alterations induced by ELS exposure. Although both males and females exhibited impairments in the short-term recognition memory task, only females exhibited deficits in working memory. These findings align with previous clinical and preclinical studies, which indicate that females are at greater risk of ELS [38–40]. Despite being highly significant, the underlying mechanisms contributing to the heightened vulnerability of females to ELS have not been fully elucidated. Moreover, this could also be attributed to the distinct susceptibilities of certain brain regions to ELS. Working memory, which is affected only in females, relies heavily on PFC function [41], while object recognition is also dependent on connectivity between the PFC and hippocampus [42]. Although both regions are known to be stress sensitive, it is plausible that PFC-dependent functions experience sex-specific effects due to ELS exposure.

Considering the key role of the PFC in working and short-term memory function, we focused our transcriptomic analysis on this region. The majority of DEGs (13) were identified in females, while 4 DEGs were identified in males. To support the findings from the RNA-seq analysis, we selected 10 genes for qPCR validation in a separate cohort of animals. Among these genes, 9 were consistent with the transcriptomic data. The more pronounced DEGs observed in females are in line with our behavioral data, which revealed greater phenotypic deficits in females. While few studies have performed transcriptomic analysis of the PFC following ELS in both sexes [13, 14], it is possible to hypothesize that the greater number of DEGs in females could be attributed to increased vulnerability to ELS exposure.

The 70-kDa heat shock protein (HSP70)-related genes (*Hspa1b, Hspb1, and Hspa5*) were among the top upregulated candidates according to our RNA-seq results for females. HSP70s are molecular chaperones that play crucial roles in maintaining cellular homeostasis and are known to be upregulated after exposure to various stressors [43]. In accordance with our sequencing data, it has been previously reported that MS exposure increases the expression of *Hspa1b and Hspb1* in the mPFC of rats [44]. Our GO analysis indicated enrichment of chaperone-mediated protein folding and refolding processes, which are among the main functions of HSP70s. The upregulation of HSP70s occurs when misfolded proteins accumulate to refold incorrectly folded proteins, which is a hallmark of diseases associated with memory/cognitive impairments [45, 46]. *Sdf2l1,* another gene observed in our analysis*,* acts by increasing the time required for misfolded proteins to achieve a correctly folded conformation, which facilitates chaperone-mediated protein refolding processes [47]. Interestingly, we observed an upregulation of *Creld2* in females exposed to ELS. Its localization within the endoplasmic reticulum is crucial for the protein folding process, particularly in response to cellular stress. Furthermore, in addition to the genes involved in protein folding, *Ccn2, Anxa3, Sdf2l1,* and *Homer1* are directly involved in the cellular stress response. The modulation of such genes may significantly impact critical cellular functions, including proliferation, migration, and survival.

According to the RNA-seq analysis of males, *Fkbp5,* a well-known modulator of the HPA axis, was downregulated. *Fkbp5* is a negative regulator of glucocorticoid receptor (GR) signaling and directly influences cortisol/corticosterone levels [48]. While proper HPA axis function is essential for physiological homeostasis, increased or decreased reactivity of this system can lead to an altered stress response [49]. Accordingly, we previously showed that male mice in the same ELS model exhibited a blunted corticosterone response and increased anxiety-like behavior [50]. Although the males in our study were less affected in the memory tasks evaluated and had fewer DEGs than the females were, these findings may suggest that the animals exposed to ELS remain vulnerable to certain HPA-related phenotypic alterations.

It is important to interpret our data with some limitations. Our RNA-seq analysis was concentrated on a single brain region. While exposure to stressors during early development can lead to changes across multiple brain regions, we chose to focus on the mPFC due to its direct involvement in short-term and working memory functions. Additionally, to minimize the number of animals utilized in our study, we exclusively investigated adolescent animals. This decision may limit the ability of this experiment to generalize the effects of ELS across different developmental stages. Furthermore, rather than analyzing all animals, RNA-seq was conducted on a randomly selected subset of mice. Additionally, validation of DEGs was performed on ten genes rather than all DEGs identified in our analysis. Finally, although maternal behavior may exert distinct effects on males and females, we were unable to conduct video analysis to evaluate the sex-specific effects of maternal care.

In conclusion, our study showed that ELS induces impaired PFC-dependent memory performance, with more pronounced effects observed in female animals. These phenotypic alterations may be associated with inadequate maternal care during early development. Additionally, RNA-seq analysis revealed that females exhibited greater differences following ELS exposure, particularly in terms of the influence on chaperone-mediated protein folding processes. Nevertheless, male animals exposed to ELS presented an alteration in *Fkbp5,* a key regulator of HPA axis function.

## Supplementary Information

Below is the link to the electronic supplementary material.

Supplementary file1 (DOCX 304 KB)

Supplementary file2 (XLSX 3214 KB)

Supplementary file3 (XLSX 3057 KB)

Supplementary file4 (XLSX 3764 KB)

## Data Availability

All RNA-seq code and data are available at: (https://github.com/bernardo-heberle/dcnl_rna_seq_els_vs_ct). Data for behavior and Real-time PCR will be made available upon request to the corresponding author R.G.O (rogo@clin.au.dk).

## References

[1] Norman RE et al (2012) The long-term health consequences of child physical abuse, emotional abuse, and neglect: a systematic review and meta-analysis. PLoS Med 9(11):e1001349 23209385 10.1371/journal.pmed.1001349 PMC3507962

[2] Geoffroy MC et al (2016) Child Neglect and Maltreatment and Childhood-to-Adulthood Cognition and Mental Health in a Prospective Birth Cohort. J Am Acad Child Adolesc Psychiatry 55(1):33-40e3 26703907 10.1016/j.jaac.2015.10.012

[3] Teicher MH et al (2016) The effects of childhood maltreatment on brain structure, function and connectivity. Nat Rev Neurosci 17(10):652–666 27640984 10.1038/nrn.2016.111

[4] Bunea IM, Szentagotai-Tatar A, Miu AC (2017) Early-life adversity and cortisol response to social stress: a meta-analysis. Transl Psychiatry 7(12):1274 29225338 10.1038/s41398-017-0032-3 PMC5802499

[5] Kovner R, Oler JA, Kalin NH (2019) Cortico-Limbic Interactions Mediate Adaptive and Maladaptive Responses Relevant to Psychopathology. Am J Psychiatry 176(12):987–999 31787014 10.1176/appi.ajp.2019.19101064 PMC7014786

[6] McKlveen JM et al (2019) “Braking” the Prefrontal Cortex: The Role of Glucocorticoids and Interneurons in Stress Adaptation and Pathology. Biol Psychiatry 86(9):669–681 31326084 10.1016/j.biopsych.2019.04.032

[7] Menezes J et al (2020) Maternal deprivation impairs memory and cognitive flexibility, effect that is avoided by environmental enrichment. Behav Brain Res 381:112468 31917242 10.1016/j.bbr.2020.112468

[8] Viola TW et al (2019) Acute neuroinflammation elicited by TLR-3 systemic activation combined with early life stress induces working memory impairments in male adolescent mice. Behav Brain Res 376:112221 31513829 10.1016/j.bbr.2019.112221

[9] do Prado CH et al (2016) Effects of early adolescent environmental enrichment on cognitive dysfunction, prefrontal cortex development, and inflammatory cytokines after early life stress. Dev Psychobiol 58(4):482–91 26688108 10.1002/dev.21390 PMC12878819

[10] Shi DD et al (2021) Predictable maternal separation confers adult stress resilience via the medial prefrontal cortex oxytocin signaling pathway in rats. Mol Psychiatry 26(12):7296–7307 34561611 10.1038/s41380-021-01293-w

[11] Yang Y et al (2023) Activation of D1R signaling in the medial prefrontal cortex rescues maternal separation-induced behavioral deficits through restoration of excitatory neurotransmission. Behav Brain Res 441:114287 36627054 10.1016/j.bbr.2023.114287

[12] Reshetnikov VV et al (2021) Social defeat stress in adult mice causes alterations in gene expression, alternative splicing, and the epigenetic landscape of H3K4me3 in the prefrontal cortex: An impact of early-life stress. Prog Neuropsychopharmacol Biol Psychiatry 106:110068 32810572 10.1016/j.pnpbp.2020.110068

[13] Zhang YD et al (2023) Sex-specific transcriptional signatures in the medial prefrontal cortex underlying sexually dimorphic behavioral responses to stress in rats. J Psychiatry Neurosci 48(1):E61–E73 36796857 10.1503/jpn.220147 PMC9943549

[14] Pena CJ et al (2019) Early life stress alters transcriptomic patterning across reward circuitry in male and female mice. Nat Commun 10(1):5098 31704941 10.1038/s41467-019-13085-6 PMC6841985

[15] Orso R et al (2023) A systematic review and multilevel meta-analysis of the prenatal and early life stress effects on rodent microglia, astrocyte, and oligodendrocyte density and morphology. Neurosci Biobehav Rev 150:105202 37116770 10.1016/j.neubiorev.2023.105202

[16] Perry CJ et al (2021) Sex differences in the neurochemistry of frontal cortex: Impact of early life stress. J Neurochem 157(4):963–981 33025572 10.1111/jnc.15208

[17] Bach H et al (2011) Neuronal tryptophan hydroxylase expression in BALB/cJ and C57Bl/6J mice. J Neurochem 118(6):1067–1074 21740442 10.1111/j.1471-4159.2011.07379.x PMC4222035

[18] Pena CJ et al (2017) Early life stress confers lifelong stress susceptibility in mice via ventral tegmental area OTX2. Science 356(6343):1185–1188 28619944 10.1126/science.aan4491 PMC5539403

[19] Orso R et al (2019) How Early Life Stress Impact Maternal Care: A Systematic Review of Rodent Studies. Front Behav Neurosci 13:197 31555106 10.3389/fnbeh.2019.00197 PMC6724664

[20] Antunes M, Biala G (2012) The novel object recognition memory: neurobiology, test procedure, and its modifications. Cogn Process 13(2):93–110 22160349 10.1007/s10339-011-0430-z PMC3332351

[21] Krueger F (2015) A wrapper tool around Cutadapt and FastQC to consistently apply quality and adapter trimming to FastQ files. Available online at: https://www.bioinformatics.babraham.ac.uk/projects/trim_galore/. Accessed 8 Dec 2024

[22] Andrews S (2010) FastQC: A Quality Control Tool for High Throughput Sequence Data*.* Available online at: http://www.bioinformatics.babraham.ac.uk/projects/fastqc/. Accessed 8 Dec 2024

[23] Martin M (2011) Cutadapt removes adapter sequences from high-throughput sequencing reads. EMBnet J 17(1):10

[24] Ewels P et al (2016) MultiQC: summarize analysis results for multiple tools and samples in a single report. Bioinformatics 32(19):3047–3048 27312411 10.1093/bioinformatics/btw354 PMC5039924

[25] Dobin A et al (2013) STAR: ultrafast universal RNA-seq aligner. Bioinformatics 29(1):15–21 23104886 10.1093/bioinformatics/bts635 PMC3530905

[26] Liao Y, Smyth GK, Shi W (2014) featureCounts: an efficient general purpose program for assigning sequence reads to genomic features. Bioinformatics 30(7):923–930 24227677 10.1093/bioinformatics/btt656

[27] Di Tommaso P et al (2017) Nextflow enables reproducible computational workflows. Nat Biotechnol 35(4):316–319 28398311 10.1038/nbt.3820

[28] Kurtzer GM, Sochat V, Bauer MW (2017) Singularity: Scientific containers for mobility of compute. PLoS One 12(5):e0177459 28494014 10.1371/journal.pone.0177459 PMC5426675

[29] Chen X et al (2020) Robust principal component analysis for accurate outlier sample detection in RNA-Seq data. BMC Bioinforma 21(1):269 10.1186/s12859-020-03608-0 PMC7324992 32600248

[30] Love MI, Huber W, Anders S (2014) Moderated estimation of fold change and dispersion for RNA-seq data with DESeq2. Genome Biol 15(12):550 25516281 10.1186/s13059-014-0550-8 PMC4302049

[31] Champagne FA et al (2003) Variations in maternal care in the rat as a mediating influence for the effects of environment on development. Physiol Behav 79(3):359–371 12954431 10.1016/s0031-9384(03)00149-5

[32] Champagne FA et al (2007) Natural variations in postpartum maternal care in inbred and outbred mice. Physiol Behav 91(2–3):325–334 17477940 10.1016/j.physbeh.2007.03.014

[33] Curley JP, Champagne FA (2016) Influence of maternal care on the developing brain: Mechanisms, temporal dynamics and sensitive periods. Front Neuroendocrinol 40:52–66 26616341 10.1016/j.yfrne.2015.11.001 PMC4783284

[34] Davis LK et al (2020) Modified limited bedding and nesting is a model of early-life stress that affects reproductive physiology and behavior in female and male Long-Evans rats. Physiol Behav 224:113037 32603746 10.1016/j.physbeh.2020.113037

[35] Wang D et al (2020) Systematic review and meta-analysis: effects of maternal separation on anxiety-like behavior in rodents. Transl Psychiatry 10(1):174 32483128 10.1038/s41398-020-0856-0 PMC7264128

[36] Rice CJ et al (2008) A novel mouse model for acute and long-lasting consequences of early life stress. Endocrinology 149(10):4892–4900 18566122 10.1210/en.2008-0633 PMC2582918

[37] Walker CD et al (2017) Chronic early life stress induced by limited bedding and nesting (LBN) material in rodents: critical considerations of methodology, outcomes and translational potential. Stress 20(5):421–448 28617197 10.1080/10253890.2017.1343296 PMC5705407

[38] Gonzalez-Pardo H et al (2020) Sex-Specific Effects of Early Life Stress on Brain Mitochondrial Function, Monoamine Levels and Neuroinflammation. Brain Sci 10(7):447 32674298 10.3390/brainsci10070447 PMC7408325

[39] Thomas EHX, Rossell SL, Gurvich C (2022) Gender Differences in the Correlations between Childhood Trauma, Schizotypy and Negative Emotions in Non-Clinical Individuals. Brain Sci 12(2):186 35203947 10.3390/brainsci12020186 PMC8870285

[40] Goodwill HL et al (2019) Early life stress leads to sex differences in development of depressive-like outcomes in a mouse model. Neuropsychopharmacology 44(4):711–720 30188513 10.1038/s41386-018-0195-5 PMC6372611

[41] Lara AH, Wallis JD (2015) The Role of Prefrontal Cortex in Working Memory: A Mini Review. Front Syst Neurosci 9:173 26733825 10.3389/fnsys.2015.00173 PMC4683174

[42] Chao OY et al (2022) Neuronal circuitry for recognition memory of object and place in rodent models. Neurosci Biobehav Rev 141:104855 36089106 10.1016/j.neubiorev.2022.104855 PMC10542956

[43] Zatsepina OG, Evgen’ev MB, Garbuz DG (2021) Role of a Heat Shock Transcription Factor and the Major Heat Shock Protein Hsp70 in Memory Formation and Neuroprotection. Cells 10(7):1638 34210082 10.3390/cells10071638 PMC8305005

[44] Solarz A et al (2021) A Search for Biomarkers of Early-life Stress-related Psychopathology: Focus on 70-kDa Heat Shock Proteins. Neuroscience 463:238–253 33662529 10.1016/j.neuroscience.2021.02.026

[45] Wankhede NL et al (2022) Involvement of molecular chaperone in protein-misfolding brain diseases. Biomed Pharmacother 147:112647 35149361 10.1016/j.biopha.2022.112647

[46] Sweeney P et al (2017) Protein misfolding in neurodegenerative diseases: implications and strategies. Transl Neurodegener 6:6 28293421 10.1186/s40035-017-0077-5 PMC5348787

[47] Hanafusa K, Wada I, Hosokawa N (2019) SDF2-like protein 1 (SDF2L1) regulates the endoplasmic reticulum localization and chaperone activity of ERdj3 protein. J Biol Chem 294(50):19335–19348 31624144 10.1074/jbc.RA119.009603 PMC6916475

[48] Hausl AS et al (2021) The cochaperone Fkbp5 shapes the acute stress response in the paraventricular nucleus of the hypothalamus of male mice. Mol Psychiatry 26(7):3060–3076 33649453 10.1038/s41380-021-01044-x PMC8505251

[49] Bentele UU et al (2024) The effect of cognitive reappraisal and early-life maternal care on neuroendocrine stress responses. Sci Rep 14(1):6837 38514744 10.1038/s41598-024-57106-x PMC10957921

[50] Orso R et al (2020) Maternal Separation Combined With Limited Bedding Increases Anxiety-Like Behavior and Alters Hypothalamic-Pituitary-Adrenal Axis Function of Male BALB/cJ Mice. Front Behav Neurosci 14:600766 33304248 10.3389/fnbeh.2020.600766 PMC7693708

