# Supplementary material for: Sex-Specific Effects of Early-Life Stress Exposure on Memory Performance and the Medial Prefrontal Cortex Transcriptomic Pattern in Adolescent Mice
Source: Mol Neurobiol. 2025 Mar 4;62(7):8728–37. doi: 10.1007/s12035-025-04803-x (PMC12209009; doi:10.1007/s12035-025-04803-x)
Supplement: Supplementary file 1 — Supplementary file1 (DOCX 304 KB) [file 12035_2025_4803_MOESM1_ESM.docx]

**Sex-specific effects of early life stress exposure on memory performance and medial prefrontal cortex transcriptomic pattern of adolescent mice**

**Table S1.** Primers and probes ID’s or sequences

| Gene | Sequence primer forward | Sequence primer reverse |
| --- | --- | --- |
| Pgk | TGCACGCTTCAAAAGCGCACG | AAGTCCACCCTCATCACGACCC |
| Hspa1b | GAAGTCGATGCCCTCGAACA | TTCGACGTGTCCATCCTGAC |
| Hspb1 | ATGAGTGGTCGCAGTGGTTC | GAAGTGGTTGACGTCCAGGG |
| Sdf2l1 | CTGCGCACCAGATCCGTATT | CGGTCCAATAGCAGTGCAGG |
| Anxa3 | TGGGCAGTCCGTGAGTAGAA | ACCTGATGTTCCGTGGTTGT |
| Cnn2 | AGCTGCGATCACAGACTGAC | CACGCTCCGTACACAGTTCT |
| Creld2 | GTCTCTGCGTCCTGTGGTTT | GCACAACTTTTCTGGGGCAC |
| Phf21b | GACCGCTTTCCTCACACGG | GAGCTTTGCTCGACACGGA |
| Homer1 | CCTCCGGTGTTGTTCCTCAG | GCCTCCTTACACTCCCCTCA |
| Elk1 | AGGATACGTGGGGTCATGGA | GTGAGCGGTGGGGAAACG |
| Fkbp5 | TCGGTTTGCTCAGTTGGGAT | GTCTGGCTTCTGCTCTACCC |

**
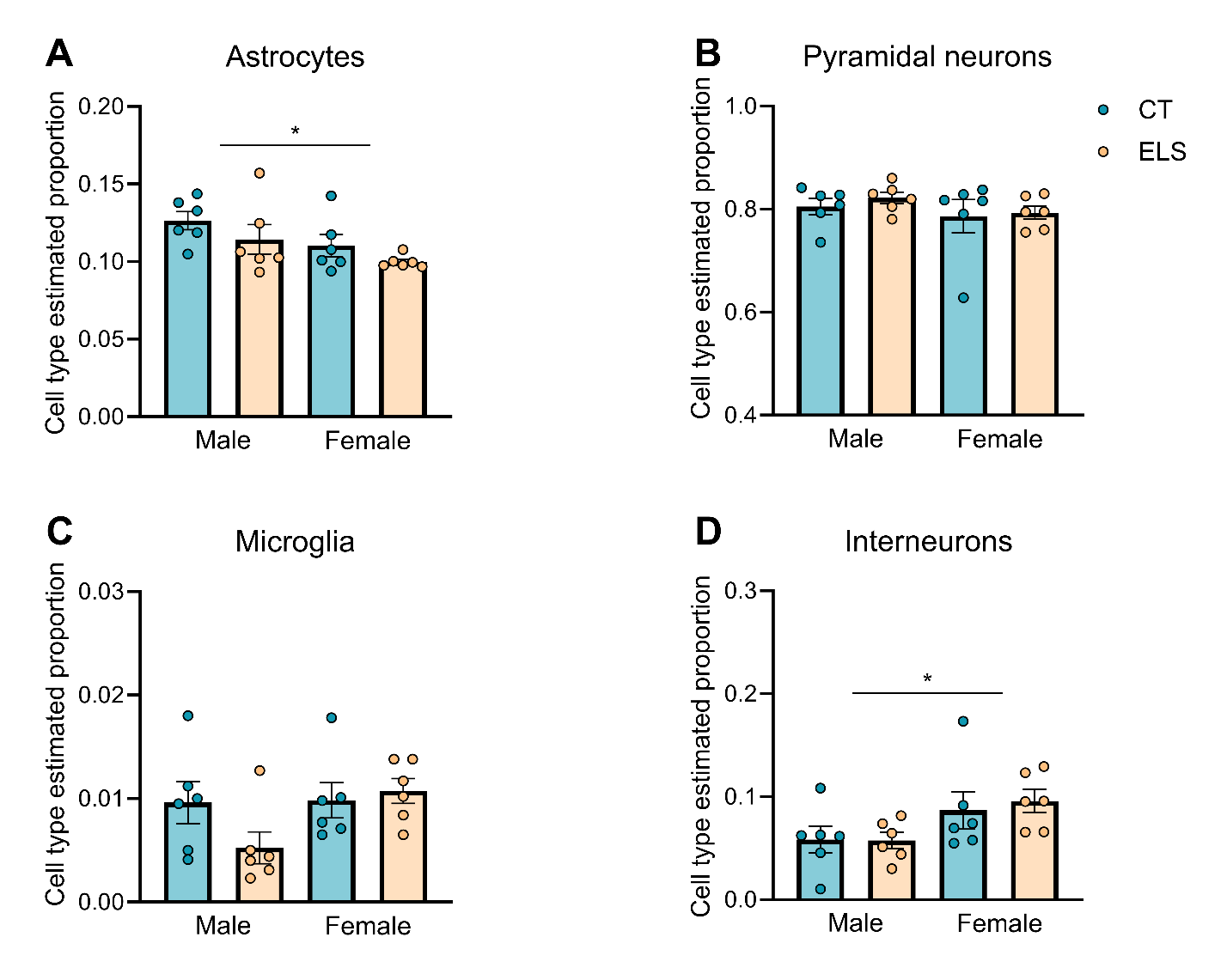
**

**Figure S1. Deconvolution of RNA-seq data.** (A-D) The proportions of Astrocytes, Pyramidal neurons, Microglia, and Interneurons were estimated in the prefrontal cortex of CT and ELS male and female mice, using single-cell RNA sequencing data as a reference. Data are expressed as mean ± SEM of 6 animals per group. *p<0.05 represents a sex effect only (Two-way ANOVA).


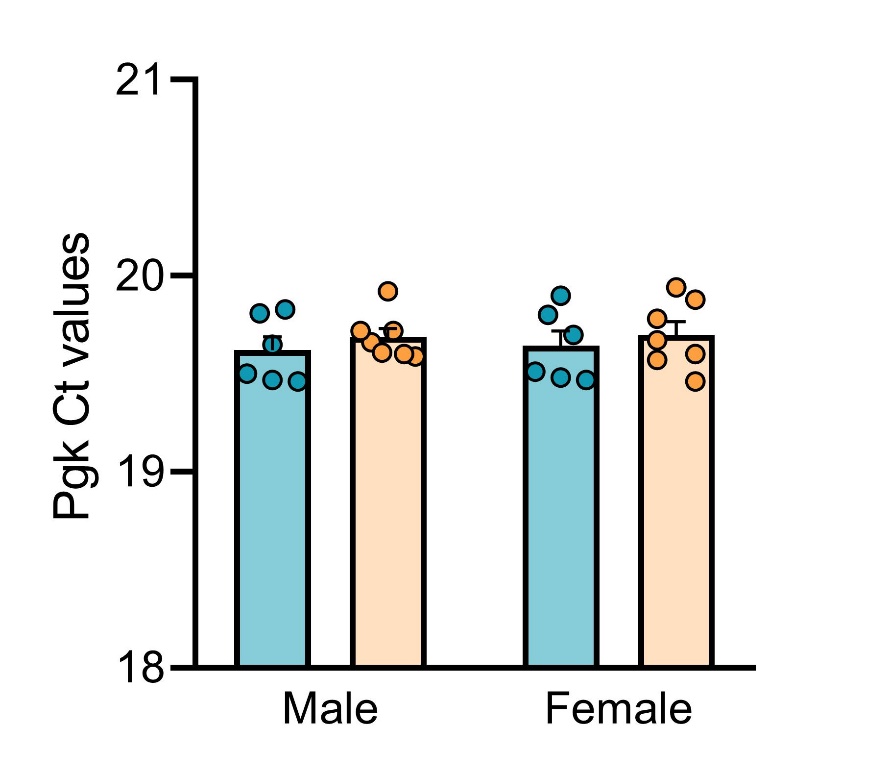


**Figure S3. Endogenous control variability**. Two-way ANOVA revealed no difference between rearing condition [F (1,22) = 0.971, p = 0.335] or sex [F (1,22) = 0.074, p = 0.786]. Cycle threshold (Ct) variation was less than 0.5 Ct among all animals included in the analysis.
